# American Proctologic Society

**Published:** 1911-09

**Authors:** 


### American Proctologic Society.

Thirteenth annual meeting, held at Los Angeles, Cal., June 26 and 27, 1911.

The President, Dr. George J. Cook of Indianapolis, Ind., in the chair.

Officers elected for the ensuing year:

President, John L. Jelks, M. D., Memphis, Tenn.

Vice-President, Alfred J. Zobel, M. D., San Francisco, Cal.

Secretary-Treasurer, Lewis H. Adler, Jr., M. D., Philadelphia, Pa.

Executive Council: George J. Cook, M. D., Indianapolis, Ind., Chairman; John L. Jelks, M. D., Memphis, Tenn.; Dwight H. Murray, M. D., Syracuse, N. Y.; Lewis H. Adler, Jr., M. D., Philadelphia, Pa.

The place of meeting for 1912 will be at Atlantic City, N. J. Exact date and headquarters to be announced later.

The following were elected Associate Fellows of the Society: Dr. Arthur F. Holding, 98 Chestnut Street, Albany, N. Y.; Dr. Ralph W. Jackson, Fall River, Mass.; Dr. E. H. Terrell, 304 East Grace Street, Richmond, Va.

The following is an abstract of some papers read:

### SOME OBSERVATIONS UPON THE SURGICAL ANATOMY AND MECHANISM OF THE COLON.

BY GRANVILLE S. HANES, M. D., OF LOUISVILLE, KY.

Until comparatively recent years diseases of the colon and sigmoid, and the surgical anatomy of each, received but scant attention. Recently, however, much valuable information upon this subject has been developed. Robert Coleman Kemp in his work on "Diseases of the Stomach and Intestines," says that Dr. J. M. Mathews was the first to call attention to sigmoiditis and diverticulitis of the sigmoid.

The entire length of the large bowel in situ is found to be much shorter than when it is dissected from its attachments. An ordinary thirty-inch colon tube has sufficient length to extend around the lumen of the large bowel to the cecum. While this has not been done in the living individual it has been done in the cadaver, and radiographs of the same are on record.

It is almost universally believed that ordinary flexible colon tubes can be manipulated in such a way as to traverse the entire course of the large bowel around to the cecum. It has been proven by a number of investigators that such an achievement is impossible in the normal bowel. The average length of the sigmoid is about eighteen inches, and this being a floating portion of the large gut it is almost impossible for an instrument to pass beyond the middle half of the sigmoid. Should such be possible and the tube enter the descending colon it would be a physical impossibility for it to pass either the acute angle at the splenic flexure or the hepatic flexure. The failure of instruments to pass high into the bowel has been demonstrated by X-ray pictures.

Dr. Hanes demonstrated the difficulty in passing any instrument through the hepatic and splenic flexures by introducing a thirty-inch, No. 20, French, soft rubber, catheter into the caput coli in an old appendicostomy case. He failed by any kind of manipulation to pass the catheter through these flexures. The tube was allowed to remain in the head of the colon for twenty-four hours with the hope that peristalsis would carry it around, but this failed. After manipulating the second time three hours later four inches of the catheter appeared through the anal opening.

He forced bismuth solution into the head of the colon till the wall of the gut was thoroughly distended, and then Dr. E. Bruce made a skiograph. No regurgitation into the ileum occurred. This experiment was repeated a number of times with the results as above given. If the ileo-cecal valve allows no reflow into the ileum then exceedingly large amounts of water injected into the bowel are retained in the large gut, and not a part of the amount passed into the small bowel as is supposed by some.

In an old appendicostomy case, with the patient on the left side, coal oil was poured into a colon tube that had been introduced three inches into the rectum. In six and a half minutes the oil was flowing out of the appendicostomy opening. The amount employed was thirty ounces. This clearly demonstrates that liquids will easily pass around the entire colon without flowing through a tube. The point is also made that coal oil is much less irritating to the mucosa than plain water or ordinary aqueous solutions.

The capacity of the large bowel in situ was measured by temporarily closing the opening of an appendicostomy case and allowing coal oil to flow into the rectum as long as the patient could tolerate it. At a later date the same experiment was made by allowing oil to flow into the head of the colon. About the same amount of oil was received in each case. After making the same experiments in other cases it was decided that the average large bowel had a capacity varying between fifty and sixty-four ounces.

The capacity of the rectum was ascertained by inverting the patient and placing a colpeurynter at the junction of the sigmoid and rectum, just within the sigmoid. The colpeurynter was then distended with air until no fluid could pass into the sigmoid. Coal oil was allowed to flow into the rectum till no more could be received. It was then drawn off with a catheter and the average amount was found to be between fourteen and seventeen ounces.

He insists that the inverted position (Hanes) is much to be preferred by both patient and operator when any kind of illuminating instruments are to be employed in the rectum or sigmoid.

### BACTERIOLOGY AND URINARY FINDINGS OF CONSTIPATION.

BY JOHN L. JELKS, M. D., MEMPHIS, TENN.

The author advances no new theories, but expresses his views of the importance of both chemical and microscopical investigation in connection with clinical proctology, and the value of these examinations in cases of atonic constipation.

He refers to the importance of either finding, or eliminating, the presence of intestinal parasites that are known to produce lesions in the intestinal coats and ports of entry of bacteria or their toxins. He expresses the belief that the destruction wrought to the submucous structures, the infiltration of plastic material and the contracting, distorting, scarred portion of the bowel, as also the consequent destruction of, and interference with the secreting glands, their ducts and the nerve supply may become important factors in the atonic condition of some patients.

The author believes it is important to make microscopic examinations in all cases of this character, both of the crude and washed specimens, and of scrapings from the intestinal wall or from any lesion found in it. . He also examines the urine chemically and microscopically, believing this important, owing to the relationship and association of diabetes, kidney insufficiency and diseases of the kidney with cases of atonic constipation.

These examinations of the urine aid in determining the proper course of treatment, especially is this true when indicanuria, casts and sometimes traces of albumen, indicate the vicarious overwork of the tired and irritated kidneys, as also the intestinal fermentation and coprostatic auto-intoxication, which results in some cases.

The author refers to the importance also of examination of the stomach contents after test meals have been given as these may furnish in some cases a clue to etiologic factors.

Blood examinations he finds quite important in determining the amount of opsonic resistance as also for finding infections in the blood, which matters by lowering the vitality may become factors in the atonic conditions which were being discussed.

---

In suitable cases excision of the tract of a fistula in ano may result in a speedy cure. Persistence of the fistula may follow the operation, but so may it follow the slow healing procedure of free incision.—*American Journal of Surgery.*

